# Contrast-Enhanced Harmonic Endoscopic Ultrasound for Diagnosis of the Aggressiveness of Pancreatic Neuroendocrine Neoplasm

**DOI:** 10.3390/diagnostics12122988

**Published:** 2022-11-29

**Authors:** Takashi Tamura, Yuto Sugihara, Hirofumi Yamazaki, Hiromu Koutani, Takaaki Tamura, Ikuhisa Tsuda, Tomoya Emori, Yuki Kawaji, Keiichi Hatamaru, Yasunobu Yamashita, Masahiro Itonaga, Reiko Ashida, Masayuki Kitano

**Affiliations:** Second Department of Internal Medicine, Wakayama Medical University, Wakayama 641-8509, Japan

**Keywords:** contrast-enhanced harmonic endoscopic ultrasound, pancreatic neuroendocrine neoplasm, aggressive pancreatic neuroendocrine neoplasm

## Abstract

The purpose of this study is to clarify the associations between the enhancement patterns on contrast-enhanced harmonic endoscopic ultrasound (CH-EUS) and the aggressiveness and prognosis of pancreatic neuroendocrine neoplasms (PanNENs). Patients who underwent CH-EUS and were pathologically diagnosed with PanNEN were included in this study. Patients were divided into three groups according to contrast-enhancement patterns on early-phase and late-phase imaging: “Group A”, vascular rich in both phases; “Group B”, vascular rich and vascular poor in early and late phases, respectively; “Group C”, vascular poor in both phases. Of 39 patients, 25 were assigned to Group A, 7 to Group B, and 7 to Group C. The median overall survival was not reached in Groups A and B and was 335 days in Group C (*p* < 0.001). The 1-year survival rates were 100% in Group A, 60% in Group B, and 43% in Group C. Patients in Group C showed the shortest overall survival among the three groups. The vascular-poor pattern on late-phase CH-EUS had the highest sensitivity, specificity, and accuracy for aggressive PanNENs among the patterns analyzed on CH-EUS and CECT (84.6%, 91.7%, and 89.2%, respectively). CH-EUS is useful for the diagnosis of and predicting the prognosis of PanNENs.

## 1. Introduction

Pancreatic neuroendocrine neoplasm (PanNEN) has a frequency of 0.32–0.43 per 100,000 persons per year and accounts for about 1–2% of all pancreatic neoplasm cases [1,2,3,4]. The prognosis and treatment for PanNENs differ according to their World Health Organization (WHO) classification. The WHO 2017 and 2019 guidelines classify PanNENs according to tumor size, mitotic index, and Ki-67 index of surgical specimens [5]. The Ki-67 index is a marker for determining the proliferative potential of a given cell population. A “wait and watch” strategy is recommended for PanNENs of G1 classification, which are tumors less than 20 mm and nonfunctioning tumors without visible metastasis [6]. However, previous studies found that 30% of these tumors changed to aggressive PanNENs such as PanNENs with lymph node involvement [7,8]. The methods used to obtain pancreatic samples for the pathological evaluation of PanNEN include pancreatectomy and endoscopic ultrasound-guided fine-needle aspiration (EUS-FNA) [9], and it is reported that PanNENs can be correctly graded from tissue obtained using EUS-FNA [4]. However, it is sometimes difficult to perform a pancreatectomy or obtain a sufficient tissue sample using EUS-FNA.

Although EUS is the usual radiological method for diagnosing pancreatic neoplasms, it is often difficult to distinguish pancreatic ductal adenocarcinoma from PanNEN using only fundamental B-mode EUS [10]. In this respect, contrast-enhanced harmonic endoscopic ultrasound (CH-EUS) using second-generation contrast agents facilitates the evaluation of the intratumoral vascularity of pancreatic tumors. The CH-EUS contrast-enhancement pattern is, reportedly, useful for differentiating pancreatic ductal adenocarcinoma from PanNEN [11], with pancreatic ductal adenocarcinomas generally showing a hypoenhancement pattern, whereas PanNENs typically show a hyperenhancement pattern. However, some PanNENs show a hypoenhancement pattern on CH-EUS examination, similar to that of pancreatic ductal adenocarcinomas, and these PanNENs typically show a poor prognosis. This suggests that the contrast-enhancement patterns of PanNENs on CH-EUS may differ depending on their classification or tumor aggressiveness. The purpose of this study is, therefore, to clarify the associations between the tumor aggressiveness of PanNENs and their enhancement patterns on CH-EUS. This study newly evaluates the contrast-enhancement pattern of PanNENs on CH-EUS by dividing it into early and late phases according to time phase.

## 2. Patients and Methods

### Study Design

This retrospective single-center cohort study was approved by the institutional review boards of the Wakayama Medical University. We informed the study subjects of the outline of this study on the website or bulletin board of our hospital and provided an opportunity to decline participation.

## 3. Patients

The cohort consisted of consecutive patients diagnosed with PanNENs between October 2012 and March 2022 at Wakayama Medical University, Japan. Patients were included in the study if they (1) were 20 years of age or older and underwent CH-EUS and (2) were diagnosed with PanNEN using pathological diagnosis. All data were abstracted from medical records from which personal identifiers were redacted. 

## 4. Lesion Definitions

According to previous reports, aggressive PanNENs include Grade 3 (G3) tumors, neuroendocrine carcinoma (NEC), mixed neuroendocrine and non-neuroendocrine neoplasia (MiNEN), or any grade of tumor that had metastasis at diagnosis or during follow-up [8,11]. In resection cases, G1 and G2 tumors showing pathological invasion of the pancreatic parenchyma were also considered aggressive PanNENs. Peripancreatic pathologic invasion was defined as microscopic venous and/or lymphatic invasion identified around the peritumoral margin or fibrous capsule in surgical specimens [8,12]. Other G1 and G2 tumors were considered nonaggressive PanNENs. In the diagnosis of aggressive PanNENs, a lesion appearing as vascular poor on imaging was defined as an aggressive PanNEN according to a previous report [12]. Tumor size was determined from the pathology report in surgical cases. In nonoperated cases, the tumor size was determined from the imaging report, and the maximum value was used.

## 5. CH-EUS Equipment

CH-EUS was performed by endosonographers with experience of more than 500 EUS procedures. All patients underwent CH-EUS using a GF-UCT260 linear echoendoscope (Olympus Medical, Tokyo, Japan) connected to a Prosound α-10, ARIETTA 850 (Hitachi Aloka Medical, Tokyo, Japan), or EU-ME2 (Olympus Medical) ultrasound scanning system. Sonazoid (Daiichi-Sankyo, Tokyo, Japan) at 15 μL/kg was used as the ultrasound contrast agent.

## 6. CH-EUS Principle

**Figure 1:**
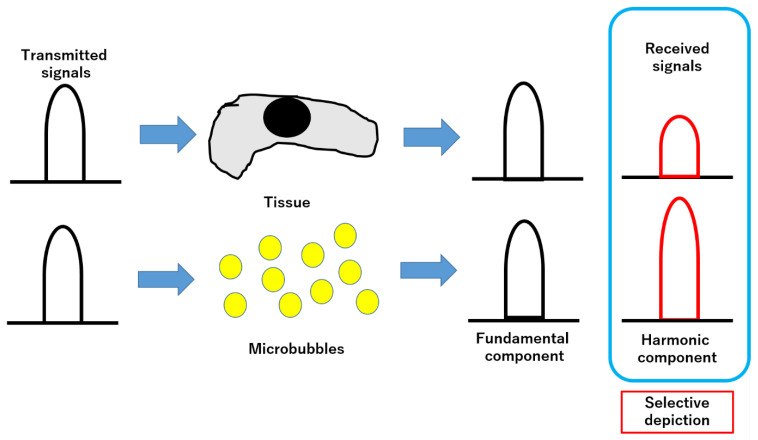
Basis of contrast harmonic imaging. When irradiated with an ultrasound beam, the microbubbles in the contrast medium vibrate. Many harmonic signals are emitted. At this time, when the transmitted ultrasound is received transmitted ultrasound is received, both the tissue and the microbubbles generates harmonic components from the microbubbles. Harmonic components from microbubbles are at higher levels than harmonic components from tissue. The harmonic components from the microbubbles are at a higher level than the harmonic components from the tissue. By selectively delineating the second harmonic component, the signal from the microbubbles is visualized more strongly than the signal from the tissue.

The ultrasound (US) contrast agent contains gas-filled microbubbles that form a lipophilic shell with a lipid monolayer. These microbubbles resonate in response to pressure waves when irradiated with US. On the other hand, the harmonic components responsible for the reflected signal from biological tissue are called “tissue harmonics”, and the reflected signal from resonant contrast agent microbubbles contains multiple insertion frequencies called harmonics [13,14,15]. However, microbubbles with large nonlinearities outweigh the tissue harmonic components. The harmonic method selectively delineates microbubbles with high sensitivity by extracting only the harmonic components [16,17]. The value of MI (mechanical index of acoustic power) that can resonate and destroy microbubbles is 0.3–0.4, allowing the contrast agent US to detect even small vessels in tissue (Figure 1) [16,17,18].

## 7. CH-EUS Procedure

First, the pancreatic mass lesion was detected on fundamental B-mode EUS, and then the viewing screen was changed to dual-screen mode with fundamental B-mode and CH-EUS mode imaging. The transmitting frequency was 5 MHz, and the MI value was 0.2–0.4. The focus point was set to the bottom of the EUS screen. The penetration of the CH-EUS ultrasound beam is inferior to that of fundamental B-mode imaging, and therefore the EUS should be positioned as close as possible to the target lesion before performing CH-EUS. Sonazoid was injected intravenously, followed by an intravenous injection of 10 mL of physiological saline.

## 8. CH-EUS Evaluation

**Figure 2:**
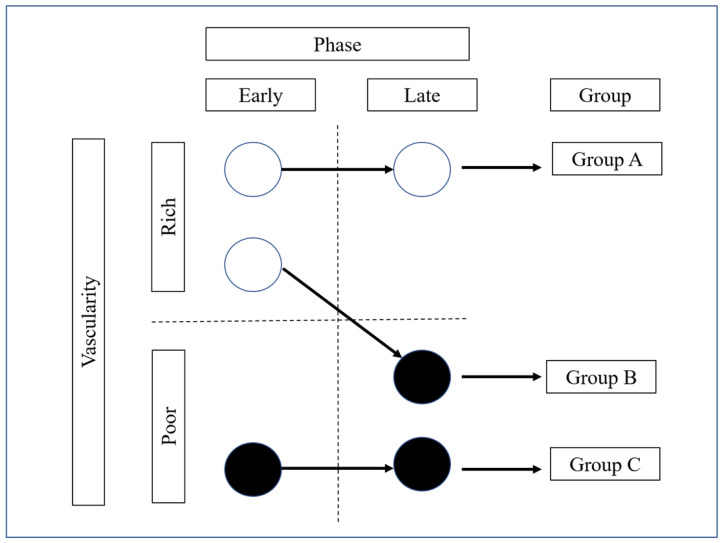
Schematic showing the evaluation of intratumoral vascularity in each phase of contrast-enhanced harmonic endoscopic ultrasound. Enhancement patterns were identified as early phase and late phase. Patients were divided into two patterns according to intratumoral vascularity: rich vascularity (iso- or hyperenhancement) and poor vascularity (hypo- or nonenhancement). Patients were finally classified into one of three potential groups according to vascularity in early and late phases, with no patients being vascular poor on early-phase imaging and vascular rich on late-phase imaging.

In a pancreatic lesion, the signal from the contrast agent appears 10–15 s after injection and reaches a peak approximately 20 s later. The examination of a lesion lasted a minimum of 90 s after the Sonazoid injection. The contrast phase was defined as the arterial phase (early) of 10–30 s (with enhancement of the abdominal aorta, celiac artery, and superior mesenteric artery), followed by the venous phase (late) of 30–90 s (with enhancement of the splenic vein, portal vein, and superior mesenteric vein). Solid pancreatic lesions were classified into four patterns according to the intensity of their enhancement on CH-EUS: nonenhancement, hypoenhancement, isoenhancement, and hyperenhancement. Then, the solid pancreatic lesions were classified into two groups based on the status of the intratumoral vessels: vascular-rich appearance (hyper- or isoenhancement) and vascular-poor appearance (non- or hypoenhancement). In addition, to evaluate lesions according to the differences in their intratumoral vascularity pattern on CH-EUS, patients were classified into the following three groups according to the vascular patterns on early- and late-phase imaging. PanNENs that had a vascular-rich appearance in both phases were defined as Group A, PanNENs that had a vascular-rich appearance on early-phase imaging and a vascular-poor appearance on late-phase imaging were classified as Group B, and PanNENs that had a vascular-poor appearance in both phases were classified as Group C (Figure 2). None of the included PanNENs were vascular poor on early-phase imaging and had a vascular-rich appearance on late-phase imaging.

All EUS images were interpreted by two endoscopists (T.T. and Y.S.) who were blinded to the clinical findings. If there was a difference in the evaluation between the two readers, the radiologists reviewed the data together until a consensus was reached.

## 9. Acquisition and Evaluation of Contrast-Enhanced Computed Tomography (CECT)

A 64-channel multidetector CT scanner (Aquilion One, Toshiba Medical Systems, Tochigi, Japan) with a section thickness of 1 mm was used for CECT. After an unenhanced scan, patients were administered nonionic contrast material (1.7 mL/kg body weight) with an iodine concentration of 350 mg/mL (Iomeron; Eisai, Tokyo, Japan) for a fixed duration of 25 s. The scan delay for the arterial and portal venous phases was 20 and 48 s, respectively, after aortic enhancement exceeded 150 HU compared with the baseline value. Pancreatic lesions were evaluated for attenuation relative to that of the surrounding pancreatic parenchyma and were classified as hypo-, iso-, or hyperdense according to the methods of a previous study [19]. The solid pancreatic lesions were also classified into two groups based on the status of the intratumoral vessels: vascular-rich appearance (hyper- or isodense) and vascular-poor appearance (non- or hypodense). Interobserver differences were resolved by consensus.

## 10. Classification and Final Diagnosis of PanNENs

PanNENs were diagnosed pathologically using hematoxylin and eosin staining and an immunohistochemical analysis of chromogranin A, synaptophysin, and/or CD56. The specimens were obtained using surgical resection or EUS-FNA. The Ki-67 proliferation index was also evaluated from the surgical specimen or EUS-FNA sampling. In the case of both a surgical specimen and an EUS-FNA sample, the surgical specimen was used. The grading of the tumors was based on the WHO 2017 classification with three groups: G1, Ki-67 < 2%; G2, Ki-67 = 3–20%; and G3, Ki-67 > 20%.

## 11. Outcomes

The primary outcomes were associations between the contrast-enhancement patterns of PanNENs on CH-EUS imaging and tumor aggressiveness, with tumor size, grade classification, and overall survival being analyzed.

Secondary outcomes were the sensitivity, specificity, positive predictive value (PPV), negative predictive value (NPV), and accuracy of CH-EUS imaging for the diagnosis of aggressive PanNENs. A further outcome is a comparison of the diagnostic values of CH-EUS and CECT for aggressive PanNENs. A subgroup analysis was performed to reveal the characteristic contrast-enhancement patterns of aggressive PanNENs of the G1 or G2 classification.

## 12. Statistical Analysis

Diagnostic values including diagnostic sensitivity, specificity, PPV, NPV, and accuracy were calculated. Among the demographic and clinical characteristics, categorical variables were analyzed using the Chi-squared test or Fisher’s exact test, while continuous variables were compared using the Wilcoxon rank-sum test or Student’s *t*-test. Prognostic ability was analyzed using the Kaplan–Meier method, and surviving cases were censored. The log-rank test was used to compare survival curves. McNemar’s test was used to compare the diagnostic accuracy of CH-EUS and CECT for progressive PanNEN.

Kappa values were calculated to assess interobserver variability in each phase and for each assessment of intratumor vascularity on CH-EUS, and 95% confidence intervals (CIs) were estimated using the jack-knife method. Kappa values were interpreted as follows: 0.00–0.20: poor agreement; 0.21–0.40: fair agreement; 0.41–0.60: moderate agreement; 0.61–0.80: substantial agreement; 0.81–1.00: almost perfect agreement. Statistical analyses were performed using JMP (Version Pro 13.0) and EzR (version 3.4.1) software. For all analyses, *p* values < 0.05 were considered to indicate statistical significance [20].

## 13. Results

### Population

The patient characteristics are summarized in Table 1. A total of 39 patients were diagnosed with PanNEN by pathological evaluation between October 2012 and March 2022. Twenty-three patients (59.0%) had G1 PanNEN, five patients (13.2%) had G2 PanNEN, two patients (5.3%) had G3 PanNEN, and five patients (13.2%) had neuroendocrine neoplasm carcinoma (NEC). Fifteen patients (38.4%) had an aggressive PanNEN, of which eight patients had an aggressive PanNEN with a G1 or G2 tumor and seven patients had an aggressive PanNEN with a G3 or NEC tumor. Five patients (12.8%) had a functioning PanNEN, fourteen patients (35.9%) underwent pancreatectomy for PanNEN, and ten patients (25.6%) received chemotherapy. One patient (2.6%) was unable to receive treatment for aggressive PanNEN. Ten patients (25.6%) underwent a wait-and-see approach for PanNEN. There was no adverse event associated with CH-EUS. The median follow-up period in the 39 patients with PanNEN was 21 months (range: 1–111 months).

## 14. Prognostication According to CH-EUS Findings

**Figure 3:**
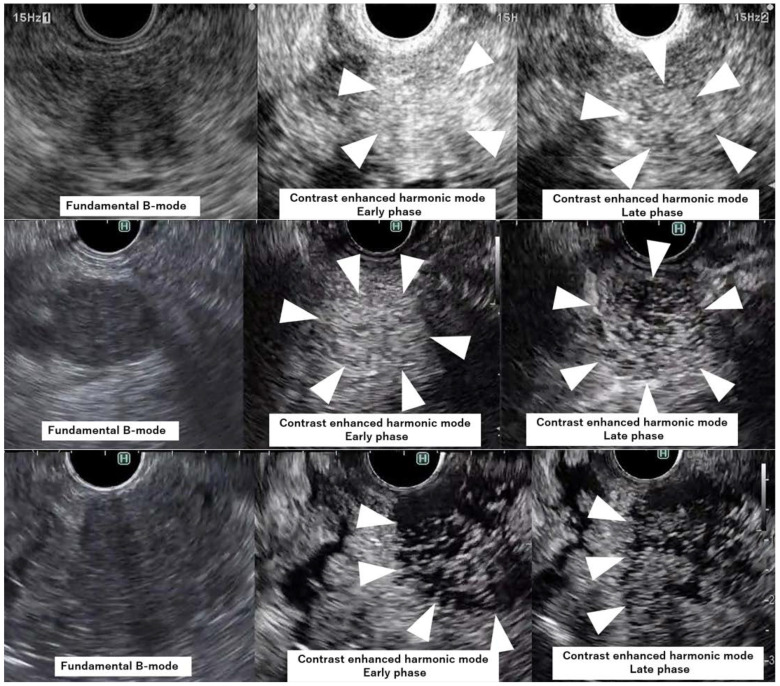
Evaluation of intratumoral vascularity on CH-EUS. A: Representative case from Group A showing a vascular-rich tumor in both phases. A pancreatic lesion was detected as a low-echoic lesion (arrowhead) on fundamental B-mode EUS (Pre). Intratumoral vascularity was evaluated as rich vascularity (arrowheads) on both early- (Early) and late-phase CH-EUS (Late). B: Representative case from Group B showing a vascular-rich and vascular-poor tumor in the early and late phases, respectively. A pancreatic lesion was detected as a low-echoic lesion (arrowheads) on FB-EUS (Pre). Intratumoral vascularity was evaluated as rich vascularity (arrowheads) on early-phase CH-EUS (Early) and poor vascularity (arrowheads) on late-phase CH-EUS (Late). C: Representative case from Group C showing a vascular-poor tumor in both phases. A pancreatic lesion was detected as a low-echoic lesion (arrowheads) on FB-EUS (Pre). Intratumoral vascularity was evaluated as poor vascularity (arrowheads) on both early- (Early) and late-phase CH-EUS (Late).

The evaluations were classified into three groups according to the vascularity in the early and late phases of CH-EUS as follows: Group A = 25 patients, Group B = 7 patients, and Group C = 7 patients (Figure 3). Kappa values were calculated to assess the interobserver variability in each phase and in each intratumoral vascularity group of CH-EUS, with a kappa value of 0.70 taken to indicate substantial agreement.

The patient characteristics of the three groups are summarized in Table 2. There are no significant differences in patient characteristics such as age, sex, tumor location, and number or function of PanNENs between the three groups. However, there are significant differences in tumor size, number of tumors under 20 mm, PanNEN grade according to WHO classification, clinical stage, and number of aggressive PanNENs (*p* = 0.004, 0.029, 0.011, 0.001, and <0.001, respectively).

**Figure 4:**
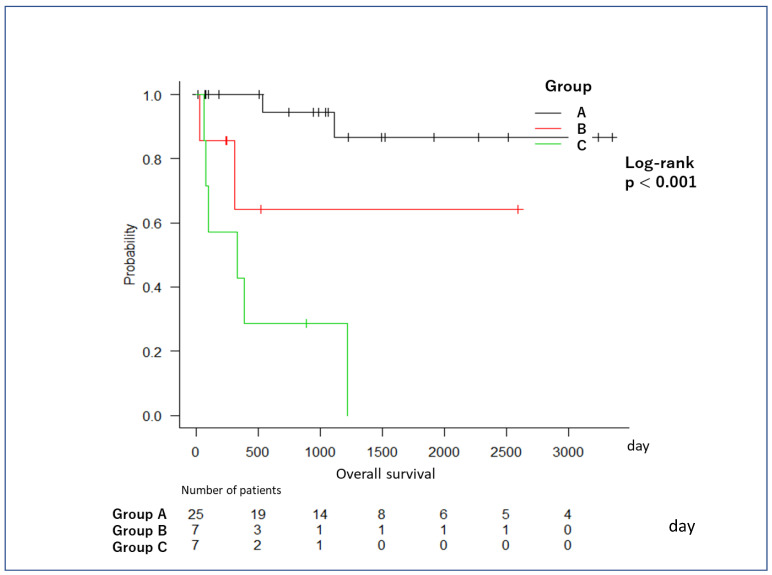
Kaplan–Meier curves showing overall survival in the three groups. There is a significant difference in overall survival between the three groups (*p* ≤ 0.001). The median overall survival was not reached in Groups A and B and was 335 days in Group C (Figure 1). Patients in Group C showed the shortest overall survival among the three groups, while patients in Group A showed the longest overall survival.

Kaplan–Meier curves showing overall survival in the three groups are presented in Figure 4. There is a significant difference in overall survival between the three groups (*p* ≤ 0.001). The median overall survival was not reached in Groups A and B and was 335 days in Group C (Figure 4). Patients in Group C showed the shortest overall survival among the three groups, whereas patients in Group A showed the longest overall survival. The 1-year survival rates were 100% in Group A, 60% in Group B, and 43% in Group C.

## 15. Comparison of the Diagnostic Values of CH-EUS and CECT for Aggressive PanNENs

Thirty-seven patients underwent both CH-EUS and CECT. Two patients could not undergo CECT because of severe renal failure. Table 3 lists the enhancement patterns of the PanNENs on CH-EUS and CECT. Table 4 shows the results of the diagnostic values of CH-EUS and CECT for aggressive PanNENs in 37 patients. For the diagnosis of aggressive PanNEN, the sensitivity, specificity, PPV, NPV, and accuracy (numbers and 95% CI) of early-phase CH-EUS are 38.5% (13.9–68.4%), 91.7% (73.0–99.0%), 71.4% (29.0–96.3%), 73.3% (54.1–87.7%), and 73.0% (55.9–86.2%), respectively. The corresponding values for late-phase CH-EUS are 84.6% (54.6–98.1%), 91.7% (73.0–99.0%), 84.6% (54.6–98.1%), 91.7% (73.0–99.0%), and 89.2% (74.6–97.0%), respectively. The vascular-poor pattern on late-phase CH-EUS has the highest sensitivity, specificity, PPV, NPV, and accuracy of aggressive PanNENs among the patterns analyzed on CH-EUS and CECT. However, there is no significant difference in the accuracy of the diagnosis of aggressive PanNENs between late-phase CH-EUS and portal-phase CECT.

## 16. Characteristics of the CH-EUS Contrast Enhancement Patterns in Eight Aggressive PanNENs with G1 or G2 Tumors

The 15 patients with aggressive PanNENs were divided into those with G1 or G2 tumors and those with G3 tumors or NEC.

Table 5 lists the contrast-enhancement patterns of the aggressive PanNENs. The contrast-enhancement patterns of the eight aggressive PanNENs with G1 or G2 tumors are as follows: Group A = 2 patients (25%), Group B = 4 patients (50%), and Group C = 2 patients (25%). In the seven aggressive PanNENs with a G3 tumor or NEC, the patterns are as follows: Group A = 1 patient (14.2%), Group B = 3 patients (42.9%), and Group C = 3 patients (42.9%). The contrast-enhancement pattern of Group B (vascular-rich appearance in the early phase and vascular-poor appearance in the late phase) was the most common contrast-enhancement pattern among the aggressive PanNENs with a G1 or G2 tumor.

## 17. Discussion

In the diagnosis of PanNENs, it is very important to predict tumor aggressiveness because some nonfunctional PanNENs with a size under 20 mm have the potential to transform into an aggressive G2–G3 tumor or a G1–G2 tumor with metastasis. The present study shows for the first time that the enhancement patterns of PanNENs on CH-EUS can be classified into three patterns and that these are useful for predicting tumor aggressiveness. On CH-EUS, PanNENs that were vascular poor in both enhancement phases showed the poorest prognosis, whereas PanNENs that were vascular rich in both phases showed the best prognosis. In addition, the present study shows that the vascular pattern on late-phase CH-EUS had the highest diagnostic value among the vascular patterns analyzed on CH-EUS and CECT.

It is reported that CH-EUS is useful not only for the diagnosis of pancreatic tumors but also for predicting their aggressiveness. Emori et al. reported that the efficacy of chemotherapy for pancreatic tumors can be predicted by evaluating intratumoral vascularity on CH-EUS [21]. The evaluation of intratumoral vascularity on CH-EUS also plays a very important role in predicting the tumor aggressiveness of PanNENs. It was reported that high intratumoral vascularity, especially microvessel density (MVD), was lower in aggressive PanNENs than in nonaggressive PanNENs [22,23]. Overproduction of the angiogenic peptide vascular endothelial growth factor (VEGF) by PanNENs may play an important role in the angiogenic process associated with PanNENs [24,25,26]. Generally, in many human tumors, the upregulation of VEGF and expression of high MVD correlate with poor clinical outcomes [27,28,29]. However, VEGF expression was found to be significantly lower in aggressive PanNENs than in nonaggressive PanNENs [23]. This is because VEGF, HiF1a, and HiF2a are strongly expressed in the cytoplasm of normal pancreatic islet cells (endocrine tissue), where they result in the presence of numerous endocrine vessels and stabilization, and this is maintained in highly differentiated tumors. However, this strong expression is disrupted in aggressive PanNEN, with VEGF, HiF1a, and HiF2a levels becoming reduced [23]. Therefore, the evaluation of the MVD of PanNENs is useful for predicting their aggressiveness.

CH-EUS and CECT are two methods that can be used to evaluate the MVD of PanNENs. In the present study, we found no significant difference in the diagnostic accuracy for PanNEN tumor aggressiveness between CH-EUS and CECT. However, a vascular-poor pattern on late-phase CH-EUS had the highest sensitivity, specificity, PPV, NPV, and accuracy of the CH-EUS and CECT patterns analyzed in the present study. Late-phase CH-EUS was more useful than early-phase CH-EUS for diagnosing aggressive PanNENs. In addition, some previous studies reported that for diagnosis of the tumor aggressiveness of PanNEN, CH-EUS had a higher accuracy than CECT and the standard criteria (size > 20 mm and/or Ki-67 proliferation index > 2%) [8,13].

The present study classifies PanNEN CH-EUS enhancement patterns into three types. In the Kaplan–Meier curves of overall survival, the patients in Group C had the shortest overall survival. The 1-year survival rate in this group was only 43%, whereas it was 60% in Group B. With respect to 1-year survival, the early phase of CH-EUS was useful for predicting the prognosis for aggressive PanNEN. The 1-year survival rate of Group C was as poor as that reported for pancreatic adenocarcinoma. The highest proportion of patients with G3 tumors or NEC was found in Group C. Takada et al. reported that G3/NEC shows weak contrast enhancement in the early-stage phase and that this weak contrast enhancement quickly declines [30]. We suggest that the vascular-poor appearance on both early- and late-phase CH-EUS in Group C may be due to the fact that the PanNENs in these patients had the most disrupted MVD among the three groups. Therefore, we suggest that CH-EUS evaluation of the MVD within PanNENs can be used to predict the prognosis of PanNENs.

CH-EUS is also a good way to evaluate the malignancy of PanNENs before surgery. The pathological evaluation of PanNENs using EUS-FNA may not always be accurate because of heterogeneity within the tumor. Therefore, treatment should be considered for PanNEN with a hypoenhancement area, even if the pathological diagnosis by EUS-FNA results in a grade of G1. If a hypoenhancement area appears within a PanNEN on follow-up CH-EUS imaging, it would be better to consider treatment for the PanNEN. It may be better to determine the treatment strategy for PanNEN with the consideration of both CH-EUS imaging and pathological evaluation using EUS-FNA.

The present study has several limitations. This is a single-center retrospective study, and the number of aggressive PanNENs is small. In 25 of 39 cases, the final diagnosis was obtained with EUS-FNA, and it was reported that for PanNENs, the agreement between grading by EUS-FNA and that by surgical specimen is approximately 84% [31]. Another limitation is that we could not evaluate the contrast enhancement of PanNENs on CH-EUS using a time-intensity curve analysis. Finally, with regard to the assessment of overall survival, it should be noted that there is no uniform treatment for aggressive PanNENs.

In conclusion, CH-EUS is useful for evaluating tumor aggressiveness and predicting the prognosis of PanNENs. Late-phase CH-EUS is useful for diagnosing aggressive PanNENs; on the other hand, early-phase CH-EUS is useful for predicting the prognosis of aggressive PanNEN. Aggressive therapeutic intervention should be considered for PanNENs that are vascular poor on CH-EUS, even for those with a tumor size of less than 20 mm and classified as Grade 1.

## Figures and Tables

**Table 1:** Patient characteristics.

Characteristic	Value
Mean age, years (range)	63 (20–83)
Sex (Male/Female)	20/19
Mean tumor diameter, mm (range)	21 (7–83)
Size >20 mm	12
Location of the tumor	
Pancreatic head	18
Pancreatic body or tail	21
Functioning tumor, n	5
Insullinoma	2
Gastrinoma	3
Final diagnosis	
Surgery	14
EUS-FNA	25
WHO classification	
G1	23
G2	5
G3	2
NEC	5
unknown	4
Mean Ki-67	
Stage, n	
I	22
II	3
III	1
IV	13
Aggressive PanNEN	15
Grade 1 or 2 with metastasis	8
Grade 3 and NEC	7
MEN type	2
Multiple PanNEN Treatment	6
Followed without surgery Surgery Endoscopic-guided ethanol injection Chemotherapy Best supportive care	13 14 2 10 1
Contrast-enhancement pattern	
Group A (vascular rich, vascular rich)	25
Group B (vascular rich, vascular poor)	7
Group C (vascular poor, vascular poor)	7

EUS-FNA—endoscopic ultrasound-guided fine-needle aspiration; NEC—neuroendocrine carcinoma; PanNEN—pancreatic neuroendocrine neoplasm; MEN—Multiple Endocrine Neoplasia.

**Table 2:** Patient characteristics according to enhancement pattern on CH-EUS.

	Group A (n = 25)	Group B (n = 7)	Group C (n = 7)	*p*-Value
Mean age, years (range)	63.0 (20–83)	61.6 (47–77)	65.4 (27–81)	0.461
Sex (Male/Female)	13/12	3/4	4/3	0.861
Mean tumor diameter, mm (range)	17.1 (7.3–87.8)	29.1 (16.3–51.6)	30.9 (7.9–74.3)	**0.004**
Size <20 mm	21	3	3	**0.029**
Size >20 mm	4	4	4	
Location of the tumor				
Head/body or tail	10/14	2/5	6/1	0.066
Functioning tumor, n				
Insullinoma	2	0	0	0.695
Gastrinoma	2	0	1	
Final diagnosis				
Surgery	11	1	1	0.144
EUS-FNA	14	6	6	
WHO classification				**0.011**
G1/G2	21	4	3	
G3/NEC	1	3	3	
Mean Ki-67 (range)	4.2 (1–33)	7.2 (1–20)	16.8 (1–40)	0.104
Stage, n				
I	20	0	2	**0.001**
II	2	1	0	
III	1	0	0	
IV	2	6	5	
Aggressive PanNEN	3	7	5	**<0.001**
Chemotherapy	1	5	4	
Followed without surgery	10	0	2	

EUS-FNA—endoscopic ultrasound-guided fine-needle aspiration; NEC—neuroendocrine carcinoma; PanNEN—pancreatic neuroendocrine neoplasm. *p*-values < 0.05 (bold) are considered statistically significant

**Table 3:** Enhancement patterns of PanNENs on CH-EUS and CECT.

			Aggressive, n	Nonaggressive, n
CH-EUS	Early phase	Vascular rich	8	22
		Vascular poor	5	2
	Late phase	Vascular rich	2	22
		Vascular poor	11	2
CECT	Arterial phase	Vascular rich	5	22
		Vascular poor	8	2
	Portal phase	Vascular rich	4	22
		Vascular poor	9	2

PanNEN—pancreatic neuroendocrine neoplasm; CH-EUS—contrast-enhanced harmonic endoscopic ultrasound; CECT—contrast-enhanced computed tomography; PPV—positive predictive value; NPV—negative predictive value.

**Table 4:** Diagnostic value of CH-EUS and CECT for aggressive PanNENs.

	Sensitivity	Specificity	PPV	NPV	Accuracy
CH-EUS					
Early phase	38.5% (13.9–68.4)	91.7% (73.0–99.0)	71.4% (29.0–96.3)	73.3% (54.1–87.7)	73.0% (55.9–86.2)
Late phase	84.6% (54.6–98.1)	91.7% (73.0–99.0)	84.6% (54.6–98.1)	91.7% (73.0–99.0)	89.2% (74.6–97.0)
CECT					
Arterial phase	61.5% (44.4–97.5)	81.5% (61.9–93.7)	61.5% (31.6–86.1)	91.7% (73.0–99.0)	81.1% (64.8–92.0)
Portal phase	69.2% (38.6–90.9)	91.7% (73.0–99.0)	81.8% (48.2–97.7)	84.6% (65.1–95.6)	83.8% (68.0–93.8)

CH-EUS—contrast-enhanced harmonic endoscopic ultrasound; CECT—contrast-enhanced computed tomography; PanNEN—pancreatic neuroendocrine neoplasm; PPV—positive predictive value; NPV—negative predictive value.

**Table 5:** Characteristics of the contrast-enhancement patterns of aggressive PanNENs on CH-EUS.

	Aggressive PanNEN of G1 or G2 (n = 8)	Aggressive PanNEN of G3 or NEC (n = 7)
CH-EUS		
Group A	2	1
Group B	4	3
Group C	2	3

CH-EUS—contrast-harmonic enhanced endoscopic ultrasound; NEC—neuroendocrine carcinoma; PanNEN—pancreatic neuroendocrine neoplasm.

## Data Availability

The datasets analyzed during the current study are available from the corresponding author upon reasonable request.
